# Angiotensin II-Induced Mitochondrial Nox4 Is a Major Endogenous Source of Oxidative Stress in Kidney Tubular Cells

**DOI:** 10.1371/journal.pone.0039739

**Published:** 2012-07-09

**Authors:** Su-Mi Kim, Yang-Gyun Kim, Kyung-Hwan Jeong, Sang-Ho Lee, Tae-Won Lee, Chun-Gyoo Ihm, Ju-Young Moon

**Affiliations:** 1 Division of Nephrology, Department of Internal Medicine, Kyung Hee University Hospital at Gangdong, College of Medicine, Kyung Hee University, Seoul, Korea; 2 Division of Nephrology, Department of Internal Medicine, Kyung Hee University, Seoul, Korea; University of Sao Paulo Medical School, Brazil

## Abstract

Angiotensin II (Ang II)-induced activation of nicotinamide adenine dinucleotide phosphate (NAD(P)H) oxidase leads to increased production of reactive oxygen species (ROS), an important intracellular second messenger in renal disease. Recent findings suggest that Ang II induces mitochondrial depolarization and further amplifies mitochondrial generation of ROS. We examined the hypothesis that ROS injury mediated by Ang II-induced mitochondrial Nox4 plays a pivotal role in mitochondrial dysfunction in tubular cells and is related to cell survival. In addition, we assessed whether angiotensin (1-7) peptide (Ang-(1-7)) was able to counteract Ang II-induced ROS-mediated cellular injury. Cultured NRK-52E cells were stimulated with 10^−6^ M Ang II for 24 h with or without Ang-(1-7) or apocynin. Ang II simulated mitochondrial Nox4 and resulted in the abrupt production of mitochondrial superoxide (O_2_^−^) and hydrogen peroxide (H_2_O_2_). Ang II also induced depolarization of the mitochondrial membrane potential, and cytosolic secretion of cytochrome C and apoptosis-inducing factor (AIF). Ang-(1-7) attenuated Ang II-induced mitochondrial Nox4 expression and apoptosis, and its effect was comparable to that of the NAD(P)H oxidase inhibitor. These findings suggest that Ang II-induced activation of mitochondrial Nox4 is an important endogenous source of ROS, and is related to cell survival. The ACE2-Ang-(1-7)-Mas receptor axis should be investigated further as a novel target of Ang II-mediated ROS injury.

## Introduction

Non-phagocytic nicotinamide adenine dinucleotide phosphate (NAD(P)H) oxidase is an enzymatic complex that plays a pivotal role in angiotensin II (Ang II)-induced reactive oxygen species (ROS) production in kidney disease. Upon stimulation with Ang II, cytosolic subunits of NAD(P)H oxidase can translocate to increase ROS production. The ROS produced after Ang II stimulation are mainly superoxide (O_2_^−^), which is converted to hydrogen peroxide (H_2_O_2_) and can also react to yield hyperchlorous acid and hydroxyl free radicals. Ang II also induces the opening of mitochondrial K_ATP_ channels, depolarizes mitochondrial potential, and further amplifies ROS generation by mitochondria, resulting in the redox-sensitive activation of mitogen-activated protein kinase (MAPK).

Nox4 is a member of the NAD(P)H oxidase complexes, which generate ROS in many cell types by transferring an electron to molecular oxygen. Recent evidence suggests that Nox4 is located on intracellular membranes in cardiac myocytes, the nucleus in vascular endothelial cells, the endoplasmic reticulum in human endothelial cells, and mitochondria in mesangial cells [1], [2], [3], [4], [5]. However, no study has examined whether Ang II stimulation of mitochondrial Nox4 is also an endogenous source of ROS in the kidney.

The recent discovery of the angiotensin-converting enzyme-related carboxypeptidase 2 (ACE2)-angiotensin-(1-7)-Mas receptor axis revealed that it has an opposing function to the ACE-Ang II-AT1 receptor axis. The angiotensin (1-7) (Ang-(1-7)) is present in kidney at concentrations comparable to those of Ang II, and is associated with vasodilation, modulation of sodium and water transport, and stimulation of nitric oxide (NO) synthase [6], [7]. Ang-(1-7) also acts as a physiological antagonist of Ang II by counter-balancing the Ang II-mediated intracellular signaling pathway. Our previous study suggested that Ang-(1-7) attenuated Ang II-mediated NAD(P)H oxidase activation and ROS production in diabetic glomeruli and mesangial cells [8].

In this study, we assessed whether Ang-(1-7) was able to counteract Ang II-induced ROS-mediated apoptosis in tubular cells. We also examined the hypothesis that ROS injury mediated by Ang II-stimulated mitochondrial Nox4 is centrally involved in mitochondrial dysfunction.

## Materials and Methods

### Cell Culture

Normal rat renal (NRK-52E) cells, which maintain the characteristics of normal renal proximal tubular cells, were purchased from the American Type Culture Collection (ATCC, Rockville, MD, USA). The renal tubular cells were cultured in Dulbecco’s modified Eagle’s medium (DMEM) supplemented with 5% fetal bovine serum (Gibco Laboratories, CA, USA), 100 U/ml penicillin, and 100 µg/ml streptomycin at 37°C in a humidified atmosphere of 5% CO_2_. The NRK-52E cells were stimulated using Ang II (10^−6^ M) with or without 20 min of pre-incubation with 10^−6^ M Ang-(1-7), A779, or apocynin (100 µmol, Sigma Chemical, MO, and Bachem Bioscience, PA, USA).

### Measurement of NAD(P)H Oxidase Activity

We determined NADPH levels as an index of redox status using a commercial colorimetric system (Biovision, CA, USA). Cultured NRK-52E cells were stimulated by Ang II with or without pre-incubation for 20 min with Ang-(1-7), A779, or apocynin. After 12 h, NADPH levels in cell lysates were measured. NADP was decomposed by heating at 60°C for 30 min. The corresponding OD450 measurements were compared with an NADPH standard curve to determine concentrations. All of the above assays were performed in triplicate dishes in at least three independent experiments.

### Intracellular ROS Detection

2′,7′-dichlorofluorescin diacetate (DCF-DA) was used to detect intracellular ROS production because the fluorescence of this cell-permeable agent significantly increases after oxidation. DCF-DA was purchased from Sigma Chemical Company and dissolved in dimethyl sulfoxide (DMSO). Cultured NRK-52E cells were stimulated by Ang II with or without 20 min of pre-incubation with Ang-(1-7) and A779. After 24 h, cells were treated with 5 µM DCF-DA for 30 min at 37°C and DCF-DA fluorescence was detected using a flow cytometer (BD FACSCalibur Flow Cytometry, USA). For image analysis of intracellular ROS, the cells were seeded on coverslips loaded into a six-well plate at a density of 2×10^5^ cells/well. NRK-52E cells were stimulated under the same conditions as those used for flow cytometry. After treatment with 30 µM DCF-DA solution at 37°C for 30 min and washing with phosphate-buffered saline (PBS), the stained cells were mounted onto microscope slides in mounting medium (DAKO, CA, USA), and images were collected using a confocal microscope (Carl Zeiss LSM 700, Oberkochen, Germany).

### Measurement of Mitochondrial Membrane Potential (Δ***Ψ***_m_)

The mitochondrial membrane potential (Δ*Ψ*_m_) of NRK-52E cells was measured using the sensitive and relatively mitochondrion-specific lipophilic cationic probe fluorochrome 5,5′,6,6′-tetrachloro-1,1′,3,3′-tetraethylbenzimidazolyl-carbocyanine iodide (JC-1) (Molecular Probes). Briefly, NRK-52E cells cultured in DMEM were incubated with JC-1 (5 µmol/L) at 37°C for 20 min and examined using flow cytometry (BD FACSCalibur). For image analysis of the generation of mitochondrial membrane potential, the cells were seeded on coverslips loaded into a six-well plate at a density of 2×10^5^ cells/well. NRK-52E cells were stimulated as they were for flow cytometry. After treatment with 5 µmol/L JC-1 for 30 min at 37°C and washing with PBS, the stained cells were mounted onto microscope slides in mounting medium (DAKO), and images were collected using a confocal microscope (Carl Zeiss LSM 700). The intensities of green (excitation/emission wavelength  = 485/538 nm) and red (excitation/emission wavelength  = 485/590 nm) fluorescence were analyzed for ≥10 microscopic fields in each sample and represented a surrogate marker of loss of mitochondrial Δ*Ψ*_m_.

**Figure 1:**
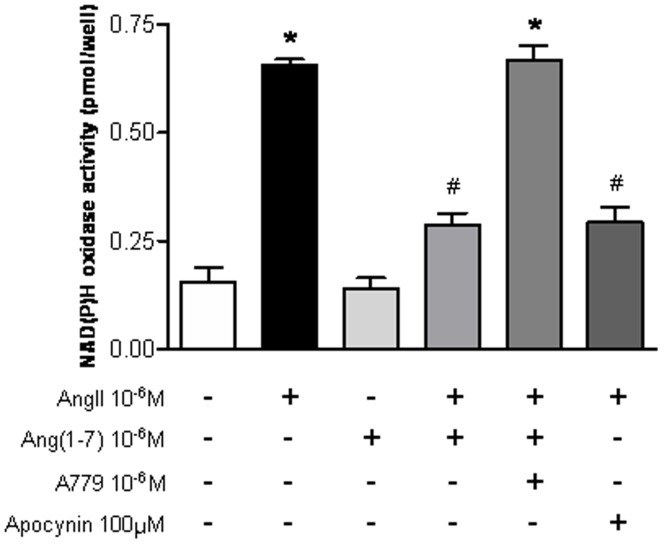
The effects of Ang-(1-7) on AngII-induced NAD(P)H oxidase activity. NAD(P)H oxidase activity was measured in NRK-52E cells treated for 24 h with Ang II (10^−6^ M), with or without Ang-(1-7) (10^−6^ M), A779 (10^−6^ M), and apocynin (100 µM). Values are the mean ± S.E.M. (n = 4). *p<0.05 versus control; ^#^p<0.05 versus Ang II-stimulated cells.

### Western Blot Analysis

Cells were washed with PBS and lysed with ice-cold lysis buffer (10 mM Tris·HCl; 150 mM NaCl; 1% Triton X-100; 5 mM EDTA, pH 8.0), and protease inhibitor cocktail (Roche Diagnostics, Mannheim, Germany). The lysate was centrifuged at 4°C for 10 min at 10,000 *g*, and the supernatant was recovered. Equal amounts of total cellular protein were subjected to SDS/PAGE in a 12% acrylamide gel, and then transferred to a polyvinylidene difluoride membrane (Millipore, Madrid, Spain) by electroblotting. The membrane was blocked with 5% fat-free milk in Tris-buffered saline with 0.5% Tween 20 (TBS-T) prior to incubation with primary antibody against Nox4, cytochrome c, prohibitin (1∶1000, Santa Cruz, CA, USA); apoptosis-inducing factor (AIF), Bax, Bcl-2, PARP, cleaved caspase-3, pan-cadherin (1∶1000, Cell Signaling Technology, MA, USA); Nox4 (1∶1000, Novus, Madrid, Spain); or Nox4 (1∶1000, Abcam, Cambridge, UK) in TBS-T and 5% bovine serum albumin (BSA) at 4°C overnight. The blots were then washed and incubated with secondary antibody in blocking solution [goat anti-rabbit horseradish peroxidase (HRP)-conjugated and goat anti-mouse HRP-conjugated, 1∶10,000; Santa Cruz] at room temperature for 1 h. The signal was detected using a pico enhanced peroxidase detection (picoEPD) Western blot detection kit (Mbiotech, Seoul, Korea), and bands were visualized using a G: Box chemi XL (Syngene, Cambridge, UK). Antibody against β-actin (1∶10,000, Santa Cruz) was used as an internal control. The results shown are representative of four blots.

### Isolation of Mitochondria (Mt)

NRK-52E cells were harvested from cultures and fractionated into cytosol (Cyt) and mitochondria using a mitochondrial isolation kit (BioChain Institute, Inc., CA, USA) according to the manufacturer’s instructions. Briefly, cells were collected, washed with 10 ml ice-cold PBS, and centrifuged at 600 *g* at 4°C for 5 min. The pellet was resuspended in 1 ml 1× mitochondria isolation buffer, transferred into a pre-chilled Dounce tissue grinder, and homogenized on ice. The homogenate was centrifuged for 10 min at 600 *g* and the collected supernatant was centrifuged for 15 min at 12,000 *g*. The resulting pellet was resuspended in 100 µl lysis buffer with 1× protease inhibitors.

### Extraction of Subcellular Membrane (Mb)

Isolation of subcellular membrane fractions was performed using the ProteoJET™ Membrane Protein Extraction Kit (Fermentas Inc., MD, USA). Briefly, the culture plate was rinsed with 4 ml of ice-cold cell wash solution; 1.5 ml cell permeabilization buffer was added to the plate and incubated for 10 min with gentle rocking. All liquid from the permeabilized cell monolayer, which contains cytoplasmic proteins, was removed, and 1 ml membrane protein extraction buffer was added to the monolayer of cells and incubated for 30 min while shaking constantly at 450 rpm. The membrane protein extract was collected into a microfuge tube and centrifuged for 15 min at 16,000 *g*. The resultant supernatant contained the membrane fraction.

**Figure 2:**
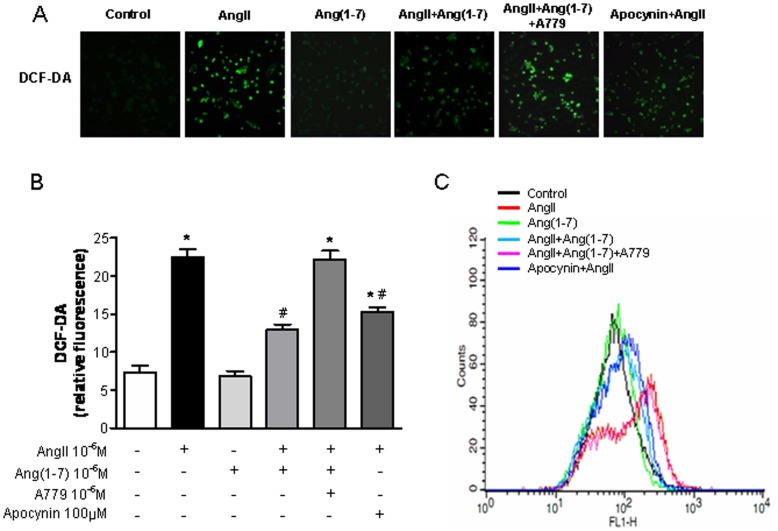
Effects of Ang-(1-7) on AngII-stimulated intracellular ROS production. A, Confocal microscopic images of cells subjected to DCF-DA treatment. Cells were exposed to Ang II (10^−6^ M) for 24 h with or without Ang-(1-7) (10^−6^ M), A779 (10^−6^ M), and apocynin (100 µM). B Measurement of DCF-DA fluorescence intensity. C, Flow cytometric analysis of cells treated with DCF-DA after treatment with Ang-(1-7) plus Ang II, reflecting a reduction in ROS generation. Values are the mean ± S.E.M. (n = 4). *p<0.05 versus control; ^#^p<0.05 versus Ang II-stimulated cells.

**Figure 3:**
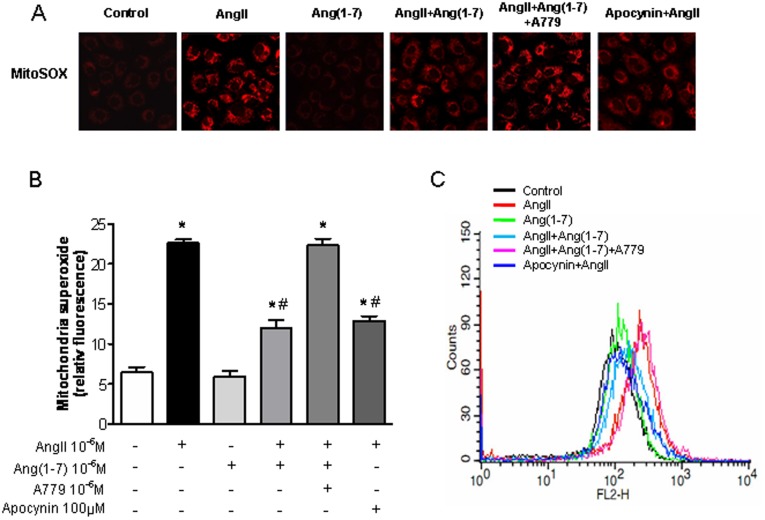
Effects of Ang-(1-7) on AngII-induced mitochondrial superoxide generation. A, Confocal microscopic images of cells subjected to MitoSOX staining. Cells were exposed to Ang II (10^−6^ M) for 24 h with or without Ang-(1-7) (10^−6^ M), A779 (10^−6^ M), and apocynin (100 µM). B, Measurement of MitoSOX fluorescence intensity. C, Flow cytometric analysis of cells treated with MitoSOX after treatment with Ang-(1-7) plus Ang II, reflecting a reduction in mitochondrial superoxide generation. Values are the mean ± S.E.M. (n = 4). *p<0.05 versus control; ^#^p<0.05 versus Ang II-stimulated cells.

### Immunocytochemistry

Cells were fixed with 4% paraformaldehyde in PBS for 1 h at room temperature and permeabilized with 1% BSA and 0.1% Triton X-100 in PBS. After blocking with 1% BSA and 0.1% Triton X-100 in PBS, the slides were incubated with primary antibodies to Nox4 and Mitotracker (Molecular Probes, OR, USA) overnight at 4°C, followed by incubation with a FITC-conjugated goat anti-mouse IgG (1∶500 dilution; Sigma-Aldrich, MO, USA) at room temperature for 2 h. The slides were mounted with VECTASHIELD HardSet Mounting Medium with DAPI, and images were acquired using a confocal microscope (Carl Zeiss LSM 700).

### Measurement of Mitochondrial Superoxide Generation

Mitochondrial superoxide generation was assessed in live cells using MitoSOX Red (Molecular Probes), a fluorogenic dye that is taken up by mitochondria, where it is readily oxidized by superoxide. NRK-52E cells were incubated with 5 µM MitoSOX Red at 37°C for 10 min and examined by flow cytometry (BD FACsCalibur). For image analysis of mitochondrial superoxide generation, the cells were seeded on coverslips loaded into a six-well plate at a density of 2×10^5^ cells/well. NRK-52E cells were stimulated as for flow cytometry. After treatment with 5 µM MitoSOX for 10 min at 37°C and washing with PBS, the stained cells were mounted onto microscope slides in mounting medium (DAKO) and images were collected by confocal microscopy (Carl Zeiss LSM 700).

### Terminal Deoxynucleotidyl Transferase dUTP Nick end Labeling (TUNEL) Assay

The number of apoptotic NRK-52E cells was measured using the TUNEL Apoptosis Detection Kit (Upstate, CA, USA), according to the manufacturer’s instructions. Briefly, after treatment the cells were fixed with 4% paraformaldehyde and incubated with a reaction mix containing biotin-dUTP and terminal deoxynucleotidyl transferase for 60 min. Fluorescein-conjugated avidin was applied to the sample and incubated in the dark for 30 min. Fluorescein-labeled cells were visualized by fluorescence microscopy and photographed.

### Annexin V Apoptosis Assays

To assess the number of apoptotic cells, we used the FITC annexin V Apoptosis Detection Kit (BD Biosciences, CA, USA) according to the manufacturer’s instructions. Briefly, after treatment the cells were washed twice with cold PBS and resuspended in 1× binding buffer at a concentration of 1×10^6^ cells/ml. One hundred microliters of the solution was transferred to a 5-ml culture tube, and 5 µl FITC annexin V and 5 µl propidium iodide (PI) were added. The cells were gently vortexed and incubated at room temperature (25°C) for 15 min in the dark before the addition of 400 µl 1× binding buffer to each tube. The cells were examined by flow cytometry (BD FACsCaliber).

**Figure 4:**
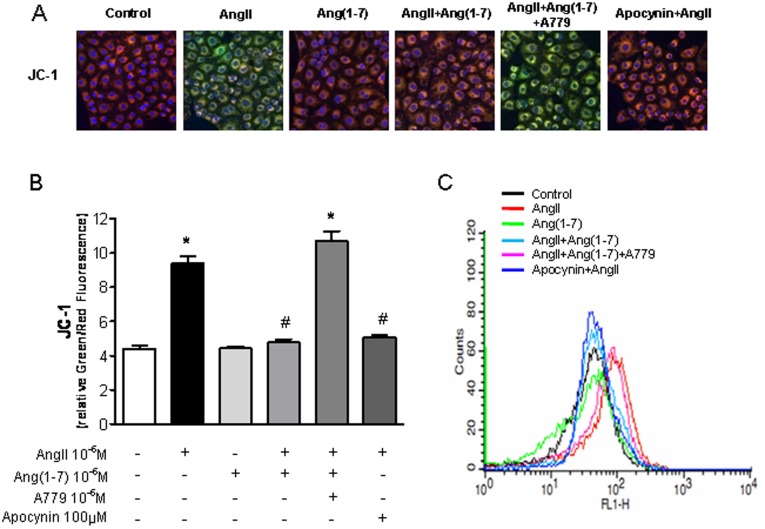
Effects of Ang-(1-7) on AngII-depolarizated mitochondrial membrane potential. A, Confocal microscopic images of cells subjected to JC-1 staining. Cells were exposed to Ang II (10^−6^ M) for 24 h, with or without Ang-(1-7) (10^−6^ M), A779 (10^−6^ M), and apocynin (100 µM). B, Measurement of JC-1 fluorescence intensity. Red staining indicates polarized mitochondria in control; green staining indicates depolarized mitochondria in Ang II with Ang-(1-7) and A779 in JC-1 staining. C, Flow cytometric analysis of cells treated with JC-1. Treatment with Ang-(1-7) rescued AngII-indued mitochondrial depolarization. Values are the mean ± S.E.M. (n = 4). *p<0.05 versus control; ^#^p<0.05 versus Ang II-stimulated cells.

### Small Interfering RNA Knockdown Experiments

Duplex small interfering RNAs (siRNAs) targeting Nox4 and a control siRNA were purchased from Bioneer Inc. (Seoul, Korea). The siRNA sequence used to target Nox4 was 5′-CAC AGU CCU GGC UUA CCU U (dTdT)-3′ (sense) and 5′-AAG GUA AGC CAG GAC UGU G (dTdT)-3′ (antisense). NRK-52E cells were transfected using 20 nM siRNA mixed with Lipofectamine 2000 (Invitrogen, CA, USA). Knockdown efficiency was assessed by Western blot analysis using anti-Nox4 and anti-β-actin antibodies, and by immunocytochemistry using anti-Nox4 antibodies. Cells were used for functional studies 24 to 72 h after transfection.

### Statistical Analysis

All values are expressed as means ± SE. Results were analyzed using the Kruskal-Wallis nonparametric test for multiple comparisons. Significant differences were confirmed by the Wilcoxon rank sum and Mann-Whitney tests to compare mean differences; p-values <0.05 were considered statistically significant.

**Figure 5:**
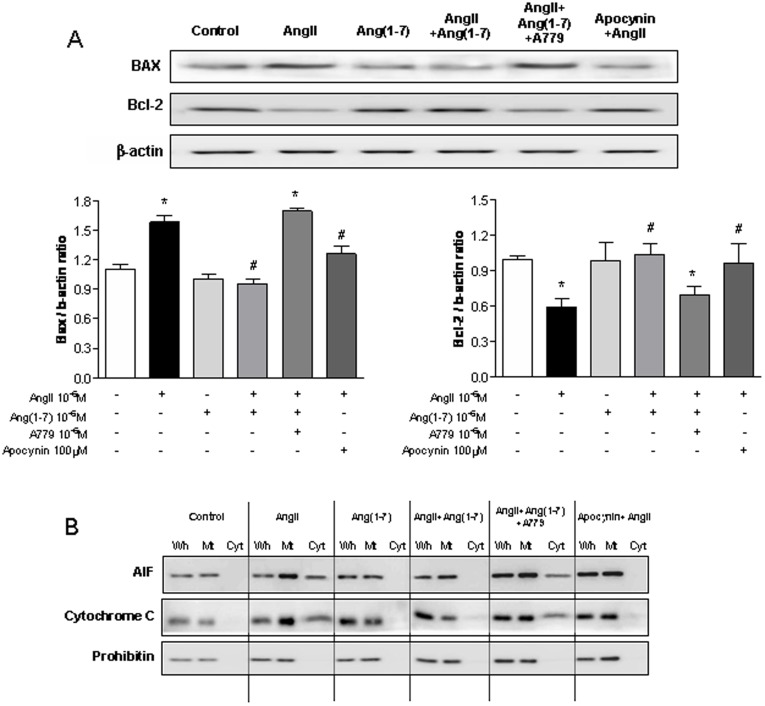
Effects of Ang-(1-7) on AngII-induced release of mitochondrial molecules. A,.Western blotting of Bax and Bcl-2 in the mitochondrial fraction and cytosolic fraction of NRK-52E cells. B,C, Above the graph are representative images of Western blots analysis each BAX and Bcl-2. D, Western blotting of AIF and cytochrome c in the mitochondrial fraction and cytosolic fraction of NRK-52E cells. All cells were exposed to Ang II (10^−6^ M) with or without Ang-(1-7) (10^−6^ M), A779 (10^−6^ M), and apocynin (100 µM). Pre-incubation with Ang-(1-7) attenuated Ang II-induced release of mitochondrial molecules. Values are the mean ± S.E.M. (n = 4).

**Figure 6:**
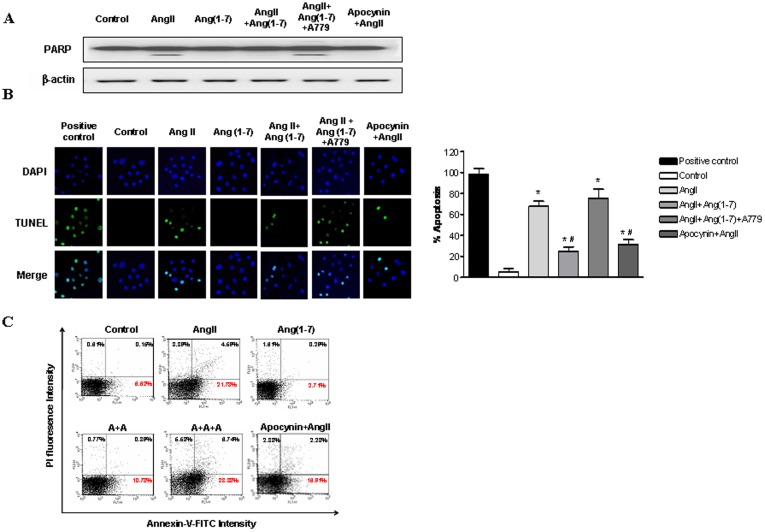
Effects of Ang-(1-7) on AngII-induced ROS-related apoptosis. A, Western blotting of PARP and cleaved caspase-3 in NRK-52E cells exposed Ang II (10^−6^ M) with or without Ang-(1-7) (10^−6^ M), A779 (10^−6^ M), and apocynin (100 µM). B, TUNEL staining of NRK-52E cells. Apoptotic nuclei are depicted by green fluorescence. TUNEL-positive nuclei in control cells after DNase I treatment. Cells were exposed to Ang II (10^−6^ M) with or without Ang-(1-7) (10^−6^ M), A779 (10^−6^ M), and apocynin (100 µM). TUNEL-positive nuclei were counted from random fields per slide and expressed as the percentage of apoptotic cells (apoptotic nuclei/total nuclei ×100%). C, Flow cytometric analysis of NRK-52E cells with annexin V-FITC/PI double staining. NRK-52E cells exposed to Ang II (10^−6^ M) with or without Ang-(1-7) (10^−6^ M), A779 (10^−6^ M), and apocynin (100 µM). Values are the mean ± S.E.M. (n = 4). *p<0.05 versus control; ^#^p<0.05 versus Ang II-stimulated cells.

## Results

### Effects of Ang-(1-7) on AngII-induced NAD(P)H Oxidase Activity in Tubular Cells

ncubation of tubular cells with Ang II led to a significant increase in NAD(P)H oxidase activity (control: 1.1±0.06 vs. Ang II: 2.4±0.09 pg/well; p<0.05). Ang-(1-7) significantly decreased the Ang II-stimulated NAD(P)H oxidase activity (Figure 1). The effect of Ang-(1-7) was similar to that of the NAD(P)H oxidase inhibitor apocynin (Ang II + Ang-(1-7): 1.3±0.07 vs. Ang II + Apocynin: 1.3±0.04 pg/well; p>0.05). The effects of Ang-(1-7) were neutralized by A779, an Ang-(1-7) receptor antagonist.

**Figure 7:**
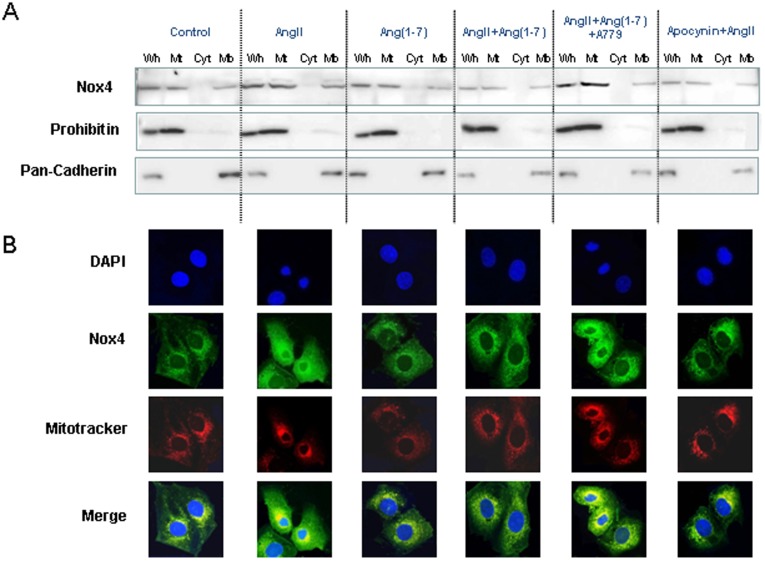
Ang II-induced mitochondrial Nox4. A, Western blotting of Nox4 in the mitochondrial fraction, subcellular membrane fraction, and cytosolic fraction of NRK-52E cells exposed to Ang II (10^−6^ M) with or without Ang-(1-7) (10^−6^ M), A779 (10^−6^ M), and apocynin (100 µM). B, Nox4 colocalizes with the mitochondrial marker Mitotracker Red FM. Values are the mean ± S.E.M. (n = 4).

**Figure 8:**
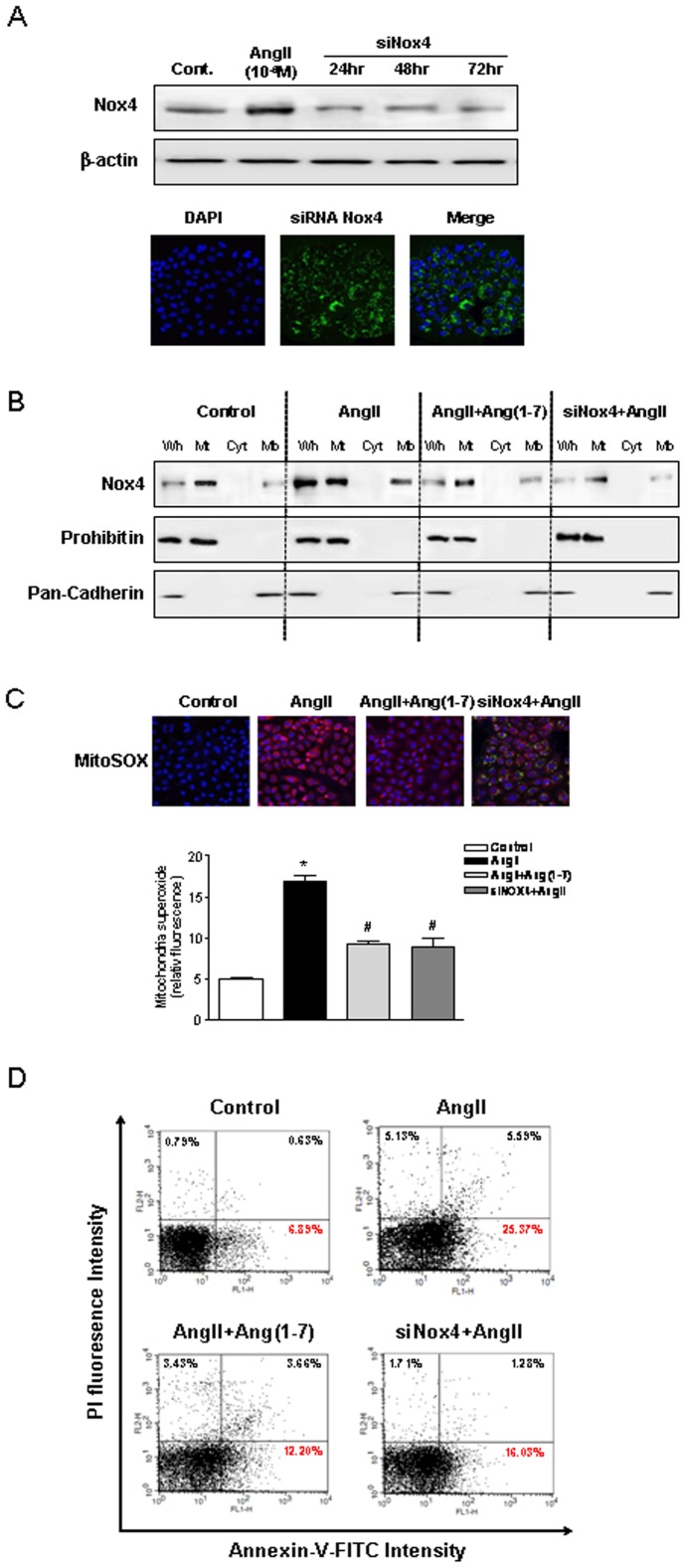
Inhibition of Nox4 by AngII attenuates mitochondrial dysfunction and apoptosis. A, Western blotting of Nox4 in the mitochondrial fraction, subcellular membrane fraction, and cytosolic fraction of NRK-52E cells. B, Confocal microscopic images of cells subjected to MitoSOX staining, and the measurement of MitoSOX fluorescence intensity. C, Flow cytometric analysis of NRK-52E cells with annexin V-FITC/PI double staining. NRK-52E cells were exposed to Ang II (10^−6^ M) with Ang-(1-7) (10^−6^ M) and transfected-siRNA Nox4. The inhibition of NOX4 was resistant to stimulation by Ang II, resulting in attenuation of mitochondrial superoxide production and inhibition of apoptosis. Values are the mean ± S.E.M. (n = 4). *p<0.05 versus control; ^#^p<0.05 versus Ang II-stimulated cells.

### Effects of Ang-(1-7) on AngII-stimulated Intracellular ROS Production

DCF-DA fluorescent dye was used to investigate the effect of Ang-(1-7) on ROS production after Ang II stimulation, in concert with confocal microscopy and flow cytometry. Confocal microscopic analysis showed that DCF-DA fluorescence markedly increased after 24 h stimulation of Ang II. Ang-(1-7) reduced the intensity of green fluorescence induced by Ang II, reflecting a reduction in ROS generation (Ang II: 23.0±0.67 vs. Ang II + Ang-(1-7): 13.5±0.45; p<0.05; Figure 2A). The effect of Ang-(1-7) was similar to that of apocynin, and was neutralized by A779. Flow cytometric analysis showed that the peak of the signal moved to the right, corresponding to increased DCF-DA fluorescence, after incubation with Ang II. This increase in fluorescence intensity was markedly reduced, as evidenced by a leftward shift after treatment with Ang-(1-7) in addition to Ang II, which reflected a reduction in ROS generation (Figure 2C).

### Effects of Ang-(1-7) on AngII-induced Mitochondrial Superoxide Generation

We confirmed the presence of mitochondrial superoxide to evaluate the source of Ang II-induced ROS. MitoSOX Red fluorogenic dye was used to selectively detect mitochondrial superoxide. Mitochondrial superoxide increased abruptly after Ang II incubation. The increase of fluorescence intensity was markedly reduced by incubation with Ang-(1-7) in addition to Ang II (Ang II: 44.5±3.3 vs. Ang II + Ang-(1-7): 27.4±2.9; p<0.05; Figure 3A, B). The effect of Ang-(1-7) was similar to that of apocynin and was neutralized by A779. Flow cytometric analysis showed that the signal moved rightward, indicating increased MitoSOX red fluorescence, after treatment with Ang II. This increase in fluorescence intensity was markedly reduced by treatment with Ang-(1-7) in addition to Ang II, as shown by a leftward shift in the signal, reflecting a reduction in mitochondrial superoxide generation (Figure 3C).

### Effects of Ang-(1-7) on Ang II-induced Depolarization of Mitochondrial Membrane Potential

To evaluate whether increased production of mitochondrial superoxide by Ang II leads to mitochondrial dysfunction, the effect of Ang II on mitochondrial membrane potential was evaluated by confocal microscopic analysis of JC-1 staining. Depolarized regions of mitochondria are indicated by the green fluorescence of JC-1 monomers. Ang II induced depolarization of the mitochondrial membrane potential, resulting in an increase in green fluorescence (and a decrease in orange fluorescence); this Ang II-induced depolarization was rescued by Ang-(1-7) (Ang II: 9.4±0.4 *vs.* Ang II + Ang-(1-7): 4.8±0.2, p<0.05; Figure 4A, B). Flow cytometric analysis revealed a rightward shift in the signal, indicating increased JC-1 green fluorescence, after Ang II treatment. This increase in fluorescence intensity was markedly reduced, and the signal shifted leftward, by treatment with Ang-(1-7) in addition to Ang II (Figure 4C), suggesting that AngII-attenuated mitochondrial function/integrity was recovered in the presence of Ang-(1-7).

### Effects of Ang-(1-7) on AngII-induced Release of Mitochondrial Molecules

We endeavored to evaluate whether depolarization of the mitochondrial membrane potential leads to the release of proapoptotic proteins from the mitochondrial intermembrane space. Tubular cells cultured with Ang II for 24 h showed a significant increase in Bax protein expression and a decrease in Bcl-2 expression (Figure 5A). However, pre-incubation with Ang-(1-7) in addition to Ang II resulted in reduced Bax expression and increased Bcl-2 expression. Ang II induced the release of AIF and cytochrome c into the cytosolic fraction; these effects were attenuated by pre-incubation with Ang-(1-7) in addition to Ang II (Figure 5B). The effect of Ang-(1-7) was similar to that of apocynin, and co-incubation with A779 neutralized the effect of Ang-(1-7).

### Effects of Ang-(1-7) on AngII-induced Apoptosis

To determine whether Ang II is involved in apoptotic cell death, we evaluated PARP by Western blot analysis (Figure 6A) and DNA fragmentation by TUNEL assay (Figure 6B) after Ang II stimulation of tubular cells. Pre-incubation of tubular cells with 10^−7^ M Ang-(1-7) in addition to Ang II significantly inhibited Ang II-stimulated apoptosis. Measurement of annexin V/PI staining by flow cytometry also showed that Ang II induced early apoptosis (Figure 6C). Ang II-induced apoptosis was inhibited by apocynin. Pre-incubation with Ang-(1-7) also attenuated Ang II-induced apoptosis.

### AngII-induced Mitochondrial Nox4

Recent studies have reported that Nox4 is located in membranes as well as mitochondria in the kidney [4] We investigated whether Ang II-induced NAD(P)H oxidase activation is related to mitochondrial Nox4 in tubular cells. We divided cell fraction lysates for Western blotting. Immunoblotting using prohibitin and pan-cadherin antibodies as mitochondrial and membrane markers, respectively, showed that treatment with 10^−6^ M Ang II for 24 h induced mitochondrial and membrane Nox4 expression (Figure 7A). Pre-incubation with 10^−6^ M Ang-(1-7) in addition to Ang II significantly inhibited Ang II-induced Nox4 expression, similar to results obtained with apocynin (100 µM). The effect of Ang-(1-7) was reversed by the addition of A779 (Figure 7A). Immunofluorescence of Nox4 showed that Nox4 co-localized with the mitochondrial marker (Figure 7B).

### Inhibition of Nox4 is Resistant to Ang II-induced Mitochondrial Dysfunction and Apoptosis

Finally, we investigated the role of mitochondrial Nox4-associated Ang II-induced mitochondrial dysfunction and apoptosis in tubular cells. Tubular cells were transfected with 20 nM Nox4 siRNA for 24, 48, and 72 h, and reduction of Nox4 expression was confirmed by immunoblotting and immunofluorescence. Nox4 siRNA-transfected tubular cells were resistant to stimulation by Ang II, resulting in decreased mitochondrial superoxide production and prevention of apoptosis (Figure 8). The effects of Nox4 siRNA were similar to the effects of Ang-(1-7) in tubular cells.

## Discussion

Ang II-induced oxidative stress and ROS are pivotal in several signal transduction pathways involved in renal pathophysiology [9], [10]. Therefore, the prevention of Ang II type1 (AT1) receptor-mediated ROS is an interesting therapeutic subject. In this study, we confirmed that the NAD(P)H oxidase Nox4 is also localized in mitochondria in renal tubular cells and is an active source of superoxide production. Ang II simulates mitochondrial Nox4, resulting in the abrupt production of mitochondrial superoxide. Furthermore, Ang II-induced Nox4 plays a critical role in mediating mitochondrial dysfunction and apoptosis in tubular cells. These effects of Ang II are attenuated by pre-incubation with Ang-(1-7) or apocynin, and by the transfection of Nox4 siRNA into proximal tubular cells. To our knowledge, no study has previously documented the role of mitochondrial Nox4 in Ang II-induced ROS activation.

The Nox4 isoform was identified as a novel kidney cDNA in human and mouse by Geiszt *et al.* [11], and was designated as renal NADPH oxidase (RENOX). The human RENOX cDNA encodes a deduced 578-amino acid protein with 90% sequence identity to its mouse counterpart [12]. The protein contains six hydrophobic segments within the N-terminal region that are proposed membrane-embedded domains involved in transmembrane electron transport, in addition to sequence motifs corresponding to proposed binding sites for heme, flavin, and NADPH. The electron transfer centers of Nox4 pass electrons from NAD(P)H to oxygen to form superoxide. Mitochondrially produced superoxide is rapidly converted into H_2_O_2_, which is readily diffusible in the intracellular space. Northern blot and *in situ* hybridization analyses have shown that RENOX mRNA is highly expressed in the kidney, particularly in the proximal tubule cells of the renal cortex. Many studies have suggested that Nox4 is a major source of ROS in the vasculature and kidney, and may function under pathologic conditions [12], [13].

Recent studies indicate that Nox4 sub-localizes to membrane, nucleus, endoplasmic reticulum, and mitochondria [4], [14], [15], [16]. Block *et al.* [4] showed that NAD(P)H-dependent superoxide generation was significantly reduced in purified mitochondrial DNA isolated from siNox4-transfected mesangial cells compared with cells transfected with scrambled siRNA, and that mitochondrial Nox4 expression is increased in the kidney cortex in a rat model of diabetes. In the present study, we similarly showed that Ang II-induced mitochondrial superoxide production was attenuated in siNox4-transfected tubular cells, resulting in reduction of apoptosis under Ang II stimulation. Ang II induction of endogenous mitochondrial Nox4 appears to be centrally involved in mediating mitochondrial dysfunction because the resulting Nox4-mediated production of intracellular H_2_O_2_ facilitates the depolarization of mitochondrial membrane potential and is related to the release of AIF and cytochrome c. It was also shown to be involved in ROS-induced ROS release (RIRR), in which increased ROS leads to mitochondrial depolarization through the activation of the mitochondrial permeability transition, yielding a short-lived burst of ROS originating from the mitochondrial electron transport chain [17]. Based on the mechanisms of RIRR, ROS generated by NAD(P)H oxidase may serve as a trigger to induce the opening of mitoKATP channels leading to the mitochondrial ROS burst, which in turn mediates the effects of Ang II. Inhibition of Nox4 prevents oxidative stress by reducing H_2_O_2_ production and by inhibiting mitochondrial dysfunction. The results of the present study are consistent with a previous study showing that the cardiac-specific deletion of Nox4 in mouse heart attenuated cardiac dysfunction, fibrosis, and apoptosis in response to pressure overload [5]. Up-regulation of Nox4 also directly affects increased mitochondrial oxidative stress and consequent mitochondrial dysfunction and cell death during heart failure [18], [19]. Therefore, the possibility of controlling mitochondrial Nox4 to regulate endogenous production of ROS represents an interesting therapeutic target.

Recently, the diverse effects of Ang-(1-7) have been actively studied in many fields, including the central nervous system, cardiovascular system, and kidney disease. One beneficial influence of Ang-(1-7) appears to be associated with the reduction of ROS, particularly those induced by Ang II. Ang II-mediated ROS are important second messengers in the transcriptional effects of Ang II, and there is strong evidence that Ang II stimulates intracellular formation of superoxide by up-regulating membrane-bound NAD(P)H oxidase and facilitating formation of its subunits [20]. Gorin *et al*. reported that Nox4-derived ROS mediate Ang II-induced Akt/protein kinase B activation and protein synthesis in mesangial cells [21]. Recent studies suggest that the ACE2-Ang-(1-7)-Mas receptor pathway modulates Ang II-mediated ROS generation and transcriptional activity [20], [22], [23]. In a previous study, we found that the effect of endogenous neutralization of Ang-(1-7) with the Ang-(1-7) receptor antagonist _D_-Ala^7^-Ang-(1-7) was associated with an approximately 1.5-fold increase in NAD(P)H oxidase activity in mesangial cells treated with a combination of Ang II and Ang-(1-7). The present study provides additional evidence that Ang-(1-7) attenuates the Ang II-induced mitochondrial Nox4 activation, and shows that its effect is comparable to that of the NAD(P)H oxidase inhibitor apocynin. The inhibition of Ang II-induced mitochondrial Nox4 by Ang-(1-7) was associated with the reduction of endogenous superoxide generation and mitochondrial dysfunction. One interesting finding in this study is the observation that Ang-(1-7) alone had an insignificant effect compared with the control. This result is consistent with findings from previous studies. Ferrario *et al*. first suggested that vascular responses to Ang-(1-7) were augmented in conditions in which endogenous ROS had been stimulated by either the induction of renovascular hypertension or a low-salt diet [24], [25], [26]. These studies indicated that Ang-(1-7) may act as an endogenous inhibitor of Ang II. Administration of recombinant ACE2 had no significant effect on systolic blood pressure at different doses in normotensive mice, although serum ACE2 activity was markedly increased [27].

In the present study, we showed that Nox4 siRNA-transfected NRK-52E cells were resistant to stimulation by Ang II, resulting in the attenuation of mitochondrial superoxide production and inhibition of early apoptosis. ROS can trigger apoptosis indirectly through damage to DNA, lipids, and proteins, or directly by ROS-mediated activation of signaling molecules. Such proapoptotic signaling of ROS may occur through the activation of MAPKs, such as the phosphorylation of SAPK/JNK, ERK, and p38 [28]. At higher ROS concentrations, hydrogen peroxide can inhibit caspases, thereby leading to a switch from apoptosis to necrosis [29], [30]. In human coronary arteries, Nox4 expression correlates with total Nox activity and endothelial function [31]. Thus, Nox4 inhibition has been considered an interesting therapeutic target. At present, no specific Nox4 inhibitors exist; however, numerous compounds from both natural and synthetic sources have Nox inhibitory effects (e.g., aryliodinium and endogenous, natural, and synthesized thiol-modifying compounds were described ca. 1990) [32]. Apocynin is now used as a classic Nox inhibitor, particularly of Nox4 and Nox5, although there is presently no evidence supporting the subunit dependence of these isoforms. The opposing actions of Ang-(1-7) on Ang II are not limited to its influences on vasoconstriction. Ang-(1-7) has been shown to reverse the hypertrophic and profibrotic effects of Ang II in the heart and the vasculature, and to oppose Ang II-mediated oxidative stress and the generation of ROS [33], [34]. The effects of Ang-(1-7) on Ang II-induced ROS have mainly been studied in the cardiovascular system. The results of the present study demonstrate that Ang-(1-7) also has a notable effect, comparable to that of apocynin, against Ang II-induced Nox4 activation in tubular cells, and that this effect was neutralized by _D_-Ala^7^-Ang-(1-7). Based on this finding, we surmise that the therapeutic potential of Ang-(1-7) lies in its ability to control Ang II-induced ROS, which play a key role in the progression of chronic kidney disease.

In conclusion, we have demonstrated that Ang II-induced mitochondrial Nox4 is an active endogenous source of superoxide production in NRK-52E cells. Moreover, Ang II-induced mitochondrial Nox4 is critically involved in mediating mitochondrial dysfunction and apoptosis in proximal tubular cells. We also found that Ang-(1-7) effectively attenuates Ang II-stimulated mitochondrial Nox4. These findings suggest that the ACE2-Ang-(1-7)-Mas receptor axis should be investigated further as a novel target of kidney disease progression.
